# Empiric ablation of asymptomatic PVCs when there is greater than 20% burden but normal left ventricular function—An argument in support of catheter ablation

**DOI:** 10.1016/j.hroo.2021.02.004

**Published:** 2021-02-26

**Authors:** Frederick T. Han

**Affiliations:** Section of Cardiac Electrophysiology University of California, San Diego Cardiovascular Institute, La Jolla, California

**Keywords:** Ablation, Asymptomatic PVCs, PVC cardiomyopathy, PVC burden, PVC medical therapy

**Key Findings**

▪ Frequent (>20%) asymptomatic premature ventricular complexes (PVCs) are associated with development of a PVC cardiomyopathy and congestive heart failure.
▪ Upon presentation, patients with frequent PVCs should initiate treatment and undergo serial monitoring for the development of congestive heart failure.
▪ PVC ablation at experienced centers has an excellent success rate and can be considered as first-line therapy for appropriately selected patients.

Should premature ventricular complex (PVC) ablation be performed empirically for patients with a normal left ventricular (LV) function? Assuming that the patient has had an extensive discussion regarding the risks and benefits of ablation of PVCs, the opinion of this author is that empiric *treatment* of these PVCs is reasonable, with the caveat that initial treatment of frequent PVCs should include medical therapy.

Since most PVCs are benign, the literature for patients undergoing PVC ablation has focused on the outcomes of patients with (1) symptomatic PVCs, (2) prevention of PVC-triggered ventricular fibrillation (VF), and (3) ablation of PVCs causing a PVC-induced cardiomyopathy. Ablation of PVCs in patients with normal LV function has become a safe and effective therapy for the treatment of patients with symptomatic PVCs,^1^ and as a result, PVC ablation holds a class 1 indication for treating frequent idiopathic symptomatic PVCs.^2^ However, there is a lack of consensus in pursuing ablation therapy for patients with a high burden of *asymptomatic* PVCs and normal LV function. Owing to the favorable outcomes with symptomatic PVC ablation and the potential to prevent the development of a PVC cardiomyopathy, empiric PVC ablation for patients with a high burden of PVCs may seem reasonable in the appropriate scenarios, but we must be cautious to avoid letting our best intentions lead us to a path of overzealous treatment. This perspective will outline my point of view of treating the asymptomatic patient with a high burden of PVCs.

## Symptom assessment

When evaluating an “asymptomatic” patient with a high burden of PVCs, a careful history will commonly detect that the patient is often not asymptomatic. A common misconception among our house staff is that if a patient does not have palpitations, dizziness, shortness of breath, or chest discomfort, they are asymptomatic with regard to their PVCs. However, if assessment of the patient’s fatigue, exercise stamina, and functional capacity is considered, one will commonly find that the patient’s primary symptoms are significant functional limitations. These limitations will commonly correlate with a decline in the patient’s quality of life and overall well-being. Screening for symptoms of syncope, presyncope, and heart failure is also essential, as idiopathic PVCs can serve as a trigger for VF or sustained ventricular tachycardia. Correlation of these symptoms on an ambulatory monitor is crucial to provide specificity of the patient’s symptoms with their PVCs. A monitor that provides a minimum of 6 days of ambulatory monitoring provides a more accurate assessment of the patient’s average PVC burden.^3^

## How many PVCs are too many?

If after a careful history and physical, a patient is identified to be truly asymptomatic with their high burden of PVCs, consideration of the risk of a PVC cardiomyopathy and its treatment should be discussed with the patient. A PVC cardiomyopathy is defined as (1) LV dilatation with reduction of LV systolic function or reduction in LV systolic function superimposed on previous LV dysfunction, (2) frequent PVCs, or (3) full or partial resolution of LV systolic dysfunction with successful treatment of the PVCs. A natural question that arises from these criteria is “What PVC burden is required to cause a cardiomyopathy?” Baman and colleagues^4^ reported their findings of 57 of 174 patients with a PVC cardiomyopathy with a mean PVC (± standard deviation) burden of 33% (± 13%) vs a PVC burden of 13% (± 12%) in patients with a normal LV systolic function. A PVC burden of >24% was identified as a cutoff for identifying patients at risk of a PVC cardiomyopathy with a 79% sensitivity and 78% specificity.^4^ However, this study did identify a patient with PVC burden of 10% with a PVC cardiomyopathy^4^; this correlates with findings from other studies with a PVC burden as low as 5%–6% causing a PVC cardiomyopathy.^5^, ^6^, ^7^ This is a sobering finding, since the PVC burden has been identified as our most reliable criterion for identifying patients at risk, so proper vigilance requires monitoring patients carefully for PVC burdens of ≥5%. Part of this variability in PVC burden and its association with PVC cardiomyopathy may be other contributing factors that include male sex, asymptomatic PVCs, repetitive monomorphic ventricular tachycardia, variability of the PVC coupling interval, PVC duration >150 ms, and epicardial PVC origin.^8^, ^9^, ^10^ Despite the variability in PVC burden associated with PVC cardiomyopathy, for the purposes of this discussion we will define a high PVC burden as ≥20%.

## Caveats for management of patients with frequent PVCs

In an asymptomatic high-PVC-burden patient, education with regard to signs and symptoms of heart failure is crucial for the early detection of PVC cardiomyopathy. These patients should undergo assessment and monitoring every 6–12 months to monitor for the development of heart failure. A PVC cardiomyopathy is characterized by reduced ventricular systolic function and ventricular dilatation, with ventricular dilatation frequently preceding the development of systolic dysfunction.^11^ Thus, serial echocardiography can reliably assess for LV dilatation, LV systolic function, mitral regurgitation, and left atrial enlargement that develop with PVC cardiomyopathy. Speckle tracking with echocardiography can also be used to detect a subclinical form of PVC cardiomyopathy (LV ejection fraction [LVEF] ≥50%) by identifying a decrease in radial, circumferential, and longitudinal strain^12^^,^^13^ that precedes the findings of ventricular dilatation and systolic dysfunction.

Unfortunately, not all patients with frequent PVCs are the same, and for some individuals frequent PVCs serve as a marker of a more sinister condition. Cardiac magnetic resonance imaging (CMR) has become an important imaging modality for differentiating these patients with a high PVC burden. CMR can identify areas of ventricular fibrosis or scar that serve as a marker for an arrhythmogenic cardiomyopathy with an increased risk of adverse outcomes.^14^, ^15^, ^16^ Muser and colleagues^17^ found that 16% of patients undergoing ablation for idiopathic PVCs had concealed myocardial abnormalities detected on CMR. These myocardial abnormalities correlated with male sex, family history of sudden death/cardiomyopathy, multifocal PVCs, and PVCs with a non–left bundle branch block inferior axis morphology.^17^ Over a follow-up of 67 months, the group with myocardial abnormalities had a 29% incidence of the composite end point of sudden cardiac death, resuscitated cardiac arrest, nonfatal episodes of VF, or sustained ventricular tachycardia requiring appropriate defibrillator therapy, compared to 0.2% of the group without myocardial abnormalities.^17^ Although there is limited data in this area, for asymptomatic high-burden PVC patients with myocardial abnormalities detected with CMR, my discussions with these patients include education on the observational data of arrhythmogenic cardiomyopathies with an increased risk of adverse events, the potential of a more complicated PVC ablation with the potential need to target multiple morphologies, and consideration of a defibrillator for prevention of sudden death.^18^, ^19^, ^20^ As a result, CMR has become an invaluable tool for the differentiation of patients with idiopathic frequent PVCs and PVC patients with arrhythmogenic cardiomyopathies, and to ensure that these patients receive the appropriate therapies.

## Treatment of PVCs vs watchful waiting

With the increased risk associated with asymptomatic PVCs and the continuum of PVC burden associated with a PVC cardiomyopathy, my preference is to avoid a watchful waiting approach and to pursue treatment of the PVCs for prevention of a PVC cardiomyopathy. This discussion with the patient highlights the risk of developing heart failure should the PVCs be allowed to occur without treatment vs the potential benefits and adverse effects of treating the PVCs. Similar to the treatment of hyperlipidemia to modify the risk of future ischemic heart disease or angiotensin-converting enzyme inhibitors and angiotensin receptor blockers to reduce the complications of diabetes, I recommend initiation of medical therapy for the treatment of the frequent PVCs.^21^^,^^22^ While the data for initiation of medical therapy for preventing PVC cardiomyopathy is not as robust as the use of statins and angiotensin-converting enzyme inhibitors / angiotensin receptor blockers, initiation of medical therapy can prove invaluable because if therapy successfully decreases the PVC burden, one can subsequently assess whether the reduction in PVC burden correlated with an improvement in the patient’s well-being or prove that the patient is truly asymptomatic. Periodic assessments of adverse effects with medical therapy is also important, as antiarrhythmic drug therapy has been associated with a discontinuation rate of 10% with both short- and long-term side effects.^22^^,^^23^ In addition, the efficacy of an antiarrhythmic drug may wane over time or the reduction in PVC burden may be suboptimal for the prevention of PVC-induced cardiomyopathy.^24^

## Medical therapy vs catheter ablation

More often than not with an asymptomatic patient, when presented with the options of medical therapy vs catheter ablation, patients tend to choose medical therapy as their first-line therapy. Typically, my first-choice options are a beta-blocker or class 1C antiarrhythmic medication (flecainide, propafenone). Ling and colleagues^22^ compared the efficacy of antiarrhythmic drug therapy (metoprolol or propafenone) to radiofrequency catheter ablation of right ventricular outflow tract (RVOT) PVCs. These patients were randomly assigned to each treatment group. At 1 year after randomization, the antiarrhythmic drug therapy group (88.6%) had a significantly higher recurrence rate of PVCs compared to the ablation group (19.4%); the antiarrhythmic drug therapy group also had a 10.3% incidence of adverse effects with medical therapy, compared to a procedural complication rate of 1.8%.^22^ In contrast, Hyman and colleagues^21^ identified 20 patients with a PVC cardiomyopathy; these patients were treated with flecainide and propafenone after a mean of 1.3 ± 0.2 failed ablations. These patients exhibited a decrease in mean PVC burden of 36% ± 3.5% to 10% ± 2.4%.^21^ Coincident with the reduction in the PVC burden, the mean LVEF increased from 37.4% ± 2.0% to 49.0% ± 1.9%.^21^ As these data illustrate, while there is a variable response to medical therapy for treatment of frequent PVCs, it still has a role as a noninvasive therapy, especially for asymptomatic patients. Whether or not there was a previous ablation performed, the PVC location and the presence of a cardiomyopathy may also be contributing factors to the different results noted above. Despite the benefits of medical therapy, catheter ablation has a significantly greater success rate of eliminating PVCs without the trial-and-error process of medical therapy. Thus, at a center that has extensive experience with ventricular arrhythmia ablation, empiric first-line ablation of RVOT PVCs can be considered, assuming the patient does not wish to pursue medical therapy or verbalizes desire to avoid serial imaging and monitoring with medical therapy.

Detractors of empiric PVC ablation in the asymptomatic patient will frequently highlight the variable and sometimes prolonged time course for developing a PVC cardiomyopathy. While animal models of PVC cardiomyopathy have shown that a PVC cardiomyopathy develops within 4 weeks, the time course in humans is significantly more variable.^25^^,^^26^ Niwano and colleagues^27^ identified the occurrence of PVC cardiomyopathy at 5.4% with minimum 4-year follow-up in a cohort of 239 patients with a PVC burden ranging from 1000 to >20,000 per 24-hour period. While these data are reassuring that a PVC cardiomyopathy can be reliably detected with serial monitoring, one also has to consider the costs, resources, and time that is expended with a serial monitoring protocol and whether or not this is a viable strategy for everyone with frequent PVCs. When these factors are taken into account, invariably some patients will choose to pursue empiric PVC ablation, especially for sites expected to have a high degree of success.

If the patient develops signs or symptoms of heart failure with their frequent PVCs, I recommend electrophysiology study and ablation of the PVCs to prevent worsening heart failure and further decline in the ventricular function. After successful ablation of the PVCs, the LVEF will frequently improve, with a mean improvement of 10%–15%.^1^^,^^24^^,^^28^, ^29^, ^30^, ^31^ While the recovery of the LVEF can be gratifying to observe, the results can be disconcerting if a patient with previously normal LV systolic function does not achieve complete recovery of the LVEF. Animal models for PVC cardiomyopathy have shown that ultrastructural changes of biventricular myocardial fibrosis, derangements in calcium handling, and sympathetic neural remodeling may contribute to an accelerated pattern of PVC cardiomyopathy development in the future with a recurrent arrhythmia.^25^^,^^26^^,^^32^ Whether these maladaptive mechanisms may contribute to a potential risk of sudden death, as has been observed with a tachycardia-induced cardiomyopathy, still remains to be determined.^33^ Despite the evidence of adverse remodeling in these PVC cardiomyopathy models, the successful treatment of PVCs and the patients’ corresponding excellent long-term outcomes precludes us from concluding that urgent ablation is required in a patient with frequent asymptomatic PVCs.^1^^,^^21^^,^^22^^,^^30^

Catheter ablation of PVCs has been reported to have superior success rates at PVC suppression in comparison with antiarrhythmic drug therapy at single centers with success rates of 80%–94%.^22^^,^^24^^,^^34^ These studies reported a complication rate of up to 5.6%.^22^^,^^24^^,^^34^ A multicenter retrospective idiopathic PVC ablation cohort of 1185 patients identified an overall ablation success rate of 84%, with RVOT PVCs having the greatest success at 93% and epicardial PVCs having the lowest success at 67%.^1^ The total complication rate was 5.2%, with 2.4% and 2.8% incidence of major and minor complications, respectively.^1^ As a result, the outcomes of PVC ablation would suggest that this is a viable first-line therapy for patients with frequent PVCs. A point of caution should arise when one considers the incidence of complications from the multicenter idiopathic PVC ablation cohort. These adverse events occurred in centers with a high degree of expertise with ventricular arrhythmia ablation, and serve as a reminder that for an asymptomatic patient, the standard for minimizing adverse events is high and must be in accordance with the patient’s expectations. Failure to identify cases with multiple morphologies of PVCs or epicardial PVCs could contribute to a failed ablation or, even worse, an adverse event incongruent with the patient’s risk tolerance. However, successful PVC ablation in an asymptomatic patient with frequent PVCs provides significant gratification for prevention of a future cardiomyopathy with its associated costs and therapies.

## Conclusion

In conclusion, our success in treating symptomatic PVCs has allowed us to consider the possibility of empiric ablation for frequent asymptomatic PVCs. While the efficacy and safety of PVC ablation provides a tempting rationale to pursue ablation empirically for asymptomatic patients, the time course of PVC cardiomyopathy development and the benefits of a close monitoring strategy with serial imaging allow us to adopt a more comprehensive approach with the empiric *treatment* of PVCs as my preferred management strategy. This treatment should start with a careful assessment of the PVC symptomatology. Should the PVCs be truly asymptomatic, medical therapy with a beta-blocker and/or class 1C agent such as propafenone or flecainide should be initiated; my rationale for this approach is that medical therapy for treating asymptomatic frequent PVCs is analogous to treating asymptomatic hyperlipidemia and hypertension for prevention of future myocardial infarctions, stroke, and renal failure. Should the patient exhibit intolerance to the medication or prefer to avoid medical therapy, then an ablation for this high burden of PVCs is a reasonable option for PVC treatment. With patients who have a single morphology of frequent asymptomatic PVCs with minimal comorbidities, treating these patients with a first-line PVC ablation is a reasonable option, provided that the anticipated efficacy and safety of the procedure are within the expectations of the patient and an experienced provider. Future randomized trials for first-line ablation vs medical therapy in patients with asymptomatic frequent PVCs with adequate long-term follow-up would provide us additional data that would enhance our ability to improve the care of these patients in both the short and long term.

## Funding Sources

This research did not receive any specific grant from funding agencies in the public, commercial, or not-for-profit sectors.

## Disclosures

Frederick T. Han has received research support and lecture honoraria from Abbott.
